# Mineral Element Composition in Grain of Awned and Awnletted Wheat (*Triticum aestivum* L.) Cultivars: Tissue-Specific Iron Speciation and Phytate and Non-Phytate Ligand Ratio

**DOI:** 10.3390/plants9010079

**Published:** 2020-01-08

**Authors:** Paula Pongrac, Iztok Arčon, Hiram Castillo-Michel, Katarina Vogel-Mikuš

**Affiliations:** 1 Jožef Stefan Institute, Jamova 39, SI-1000 Ljubljana, Slovenia; iztok.arcon@ung.si (I.A.); katarina.vogelmikus@bf.uni-lj.si (K.V.-M.); 2 Laboratory for quantum optics, University of Nova Gorica, Vipavska 13, SI-5000 Nova Gorica, Slovenia; 3 European Synchrotron Radiation Facility, 38043 Grenoble, France; hiram.castillo_michel@esrf.fr; 4 Biotechnical Faculty, University of Ljubljana, Jamnikarjeva 101, SI-1000 Ljubljana, Slovenia

**Keywords:** biofortification, phytate, iron, awn, X-ray fluorescence, X-ray absorption spectrometry, phosphorus, sulphur, nicotianamine

## Abstract

In wheat (*Triticum aestivum* L.), the awns—the bristle-like structures extending from lemmas—are photosynthetically active. Compared to awned cultivars, awnletted cultivars produce more grains per unit area and per spike, resulting in significant reduction in grain size, but their mineral element composition remains unstudied. Nine awned and 11 awnletted cultivars were grown simultaneously in the field. With no difference in 1000-grain weight, a larger calcium and manganese—but smaller iron (Fe) concentrations—were found in whole grain of awned than in awnletted cultivars. Micro X-ray absorption near edge structure analysis of different tissues of frozen-hydrated grain cross-sections revealed that differences in total Fe concentration were not accompanied by differences in Fe speciation (64% of Fe existed as ferric and 36% as ferrous species) or Fe ligands (53% were phytate and 47% were non-phytate ligands). In contrast, there was a distinct tissue-specificity with pericarp containing the largest proportion (86%) of ferric species and nucellar projection (49%) the smallest. Phytate ligand was predominant in aleurone, scutellum and embryo (72%, 70%, and 56%, respectively), while nucellar projection and pericarp contained only non-phytate ligands. Assuming Fe bioavailability depends on Fe ligands, we conclude that Fe bioavailability from wheat grain is tissue specific.

## 1. Introduction

Mineral micronutrient sufficiency—a prerequisite for human well-being—can be ensured by diet diversification or consumption of mineral-dense produce [1]. In human diets, mineral micronutrients are predominantly acquired from plant-based sources, in particular staple grain [2,3]. However, most mineral micronutrients (manganese (Mn), iron (Fe), copper (Cu) and zinc (Zn)) in grain are tightly bound in phytate (myo-inositol hexakisphosphate), a phosphorus (P)-rich salt, which cannot be digested by mammals. This makes phytate-bound mineral elements poorly bioavailable and ineffectively exploited for normal body functions [4]. Furthermore, mineral density of the cereal grain has been for a long time regarded as of minor importance compared to the crop yield [5] resulting in prevalent micronutrient deficiencies in humans [6].

Efforts to increase bio-available concentrations of mineral elements in staple crops to remedy mineral micronutrient deficiencies—particularly in marginal populations—have been invested recently, and are referred to as biofortification [7,8]. Of the seven mineral elements often lacking in our diets, Fe deficiency is most widespread, affecting up to 60% of the global population [7]. However, increasing bio-available Fe concentration through the agronomic and genetic approaches in crops is challenging [9] for several reasons: (i) poor Fe availability in the soils limits uptake into plant roots [10], (ii) strict metabolic control over Fe accumulation and sequestration in plants tissues (sufficiency ranging between 50 and 150 mg Fe·kg^−1^ dry weight in leaves of crop plants [11]), (iii) removal of Fe-rich layers during the processing of staple grain [12], and (iv) poor Fe bioavailability from phytate-rich produce such as cereal grain [13].

A large degree of variation in the accumulation of Fe in grain and seeds has been found in different crops, which is not a result from just the environmental factors. For example, in bread wheat (*Triticum aestivum* L.) grain, the variation in total Fe concentration, i.e., a ratio between the minimum and maximum total Fe concentration in grain, was up to 1.76 [14], in barley (*Hordeum vulgare* L.) the variation was up to 4.5 [15], in rice (*Oryza sativa* L.) up to 10.7 in flooded conditions and up to 288 in unflooded conditions [16], in pearl millet (*Pennisetum glaucum* (L.) R. Br.) up to 4.4 [17], in chickpea (*Cicer arietinum* L.) up to 3.2, and in pea (*Pisum sativum* L.) up to 3.5 [18]. Following predominantly classic breeding strategies, the existing natural variation in Fe density has been exploited for the development of biofortified varieties within the HarvestPlus programme [8], which demonstrated, for different crops and in different populations, that consumption of Fe-biofortified crops provides significantly more bioavailable Fe.

Despite its obvious importance for human nutrition, the filling of the staple grain with Fe, and understanding tissue-specific partitioning of Fe and Fe ligands remains a poorly understood subject [19]. Most grain filling processes take place through phloem tissues, which deliver Fe remobilised from the leaves. The presence of awns (bristle such as structures extending from lemmas), exhibiting photosynthetic activity accompanied by transpiration activity in wheat [20], may therefore play a role in the grain filling. This connection has not been investigated so far. It is, however, well-accepted that the level of phloem-mobility of a mineral element significantly affects its concentration and location in the grain, with elements such as calcium (Ca), exhibiting poor phloem mobility, not easily reaching the filial tissues of the grain and mostly remaining in the pericarp (maternal) tissues of the grain [12,21,22]. Iron has intermediate phloem mobility [23,24], so relatively large concentrations (exceeding those in leaves) of Fe have been found in some filial tissues of different staple grain, particularly the aleurone and embryo with values in the range from 200 to 400 mg Fe·kg^−1^ and from 100 to 200 mg Fe·kg^−1^, respectively [12,15,25,26,27,28]. In these grain tissues, Fe was found to strongly co-localise with P [12,28,29,30]. Since approximately 80% of total P in the grain is in the form of phytate stored mainly in the aleurone cells [31], it has been inferred that the majority of Fe is bound to phytate in these tissues. Co-localisation analyses can, however, only predict potential ligands, not unambiguously determine the Fe binding environment, so conclusions must be drawn carefully when P is being used as a proxy for phytate. Using X-ray absorption near edge structure (XANES), which enables simultaneous analysis of Fe chemical form (speciation) and the type of complexing agents, it has been shown that around 80% of Fe in the whole grain of different wheat cultivars is bound to phytate, 15% to 24% as Fe^2+^ (ferrous) and 57% to 85% as Fe^3+^ (ferric) species [25]. Obtaining Fe K-edge XANES spectra of sufficiently high signal-to-noise ratio is a challenging task, with Fe concentrations typically found in grains and particularly in endosperm (<20 mg Fe·kg^−1^ in wheat [12,32], barley [15], and Tartary buckwheat (*Fagopyrum tataricum* Gaertn.) [21]), thus such studies remain scarce. One way to circumvent these technical challenges is to combine reliable Fe distribution mapping, which identifies tissues or cell-types with the largest Fe concentrations, with micro-XANES analysis. One such study, conducted on cotyledons (containing on average 187 mg Fe·kg^−1^ [21]) of Tartary buckwheat grain showed that 47% of Fe was bound to phytate, 22%, of that as Fe^2+^ and 25% as Fe^3+^, while the remaining Fe^3+^ was bound to citrate [33]. Furthermore, in wheat aleurone, modified aleurone (surrounding the crease) and in nucellar projection the micro-XANES analysis indicated that Fe was bound to phytate/citrate, phytate, and Fe-nicotianamine/Fe oxide-hydroxide, respectively [28]. However, tissue-specific Fe speciation was not resolved [28] and the XANES analysis in pericarp and embryo have not been acquired so far.

Therefore, the aim of the study was to compare mineral element composition of the awned and awnletted (those that have short or no awns) cultivars and to determine tissue-specific Fe speciation and Fe ligands in the contrasting cultivars to test the following hypotheses: (i) the presence of awns affects the mineral element composition of the wheat grain, (ii) majority of Fe is bound to phytate in different tissues of wheat grain, (iii) Fe speciation and Fe ligands across different wheat cultivars are stable, and (iv) Fe ligand profile depends on local Fe concentration.

## 2. Results

### 2.1. Total Concentrations of Mineral Elements in Whole Wheat Grain

**Figure 1:**
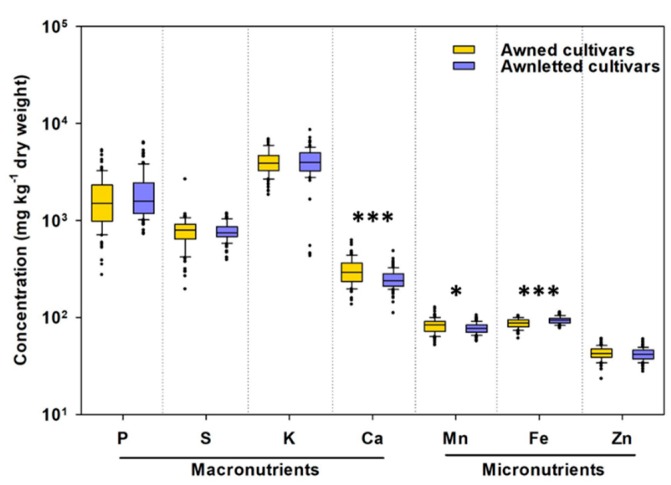
Variability in the total concentration of phosphorus (P), sulphur (S), potassium (K), calcium (Ca), manganese (Mn), iron (Fe), and zinc (Zn) in whole grain of wheat (*Triticum aestivum* L.) cultivars differing in the awn type (awned cultivars have long awns and awnletted cultivars have short or no awns) grown in the same field. Shown are boxplots representing 25th and 75th percentile of the data, with the middle line representing the median, whiskers representing the 5th and 95th percentile and the black dots representing outliers (*n* = 87 and *n* = 100 data points of nine awned and 11 awnletted wheat cultivars, respectively). Asterisks indicate significant differences between the awned and the awnletted cultivars (Student *t*-test; *** *p* < 0.001 and * *p* < 0.05).

A significantly larger total concentration of Ca and Mn, but significantly smaller total concentration of Fe was found in whole grain of awned wheat cultivars than in awnletted cultivars (Figure 1).

The total concentration of Fe in the awned wheat cultivars ranged from 50.8 to 94 mg·kg^−1^ dry weight (1.85-fold variability) and in the awnletted wheat genotypes from 67.3 to 103 mg·kg^−1^ dry weight (1.53-fold variability). Considering all wheat cultivars studied, total Fe concentration in whole grain varied 2.03-fold.

There was no difference in 1000-grain weight (Table S1) and in concentrations of P, sulphur (S), potassium (K) and Zn in the whole grain between the awned and the awnletted wheat cultivar group (Figure 1), nor was there any apparent separation of awned and awnletted cultivars when the whole elemental profile was considered (Figure S1). The hierarchical clustering indicated that Fe and Zn were grouped apart from the rest of the mineral elements, among which P, K, and S grouped apart from Ca and Mn (Figure S1). A significant positive correlation was observed between grain concentrations of P and those of K, S, and Fe, but no significant correlation between grain concentrations of P and Ca, Mn, and Zn (Figure S2a). Positive correlation was found between grain concentration of Fe and Mn, and of Fe and Zn (Figure S2b).

**Figure 2:**
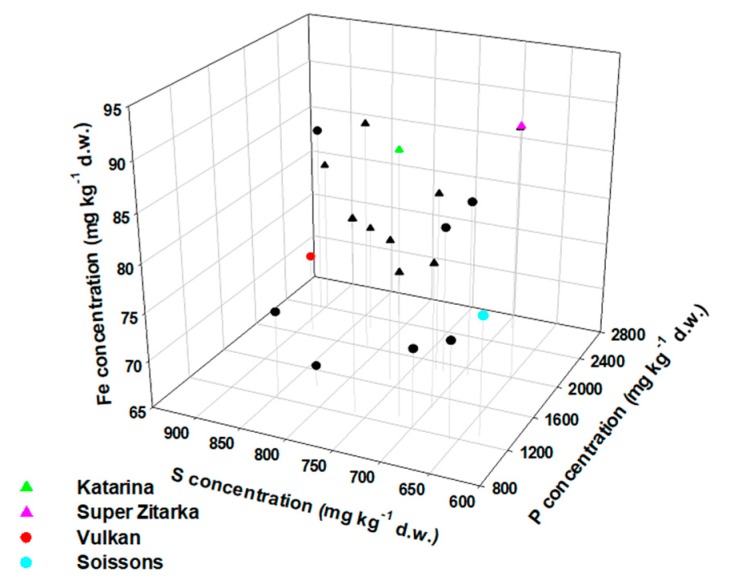
Total iron (Fe), sulphur (S) and phosphorus (P) concentrations in the grain of wheat (*Triticum aestivum* L.) cultivars differing in the awn type (awned cultivars have long awns and awnletted cultivars have short or no awns) grown in the same field. Circles represent awned cultivars and triangles represent awnletted cultivars. The four cultivars (Katarina, Super Zitarka, Vulkan and Soissons) selected for further in-depth analyses are highlighted in colour. Shown are averages (*n* = 6–12). d.w.—dry weight.

Four wheat cultivars with contrasting Fe concentrations were selected for further in-depth analyses: Two awned cultivars (cv. Vulkan and cv. Soissons) of low-Fe accumulation (the average total concentrations in the grain was 73.4 and 77.0 mg Fe·kg^−1^ dry weight, respectively), and two awnletted cultivars (cv. Katarina and cv. Super Zitarka) of high-Fe accumulation (the average total Fe concentrations in the grain was 83.7 and 91.3 mg Fe·kg^−1^ dry weight, respectively; Figure 2). In agreement with observations for all wheat cultivars studied, there was a positive correlation between grain P concentrations and grain S, K, and Fe concentration (Figure S3a) and between grain Fe and grain Zn (but not Mn) concentrations (Figure S3b) in these four wheat cultivars.

### 2.2. Tissue-Specific Iron, Phosphorus and Sulphur Concentrations, Iron Speciation and Iron Ligands

**Figure 3:**
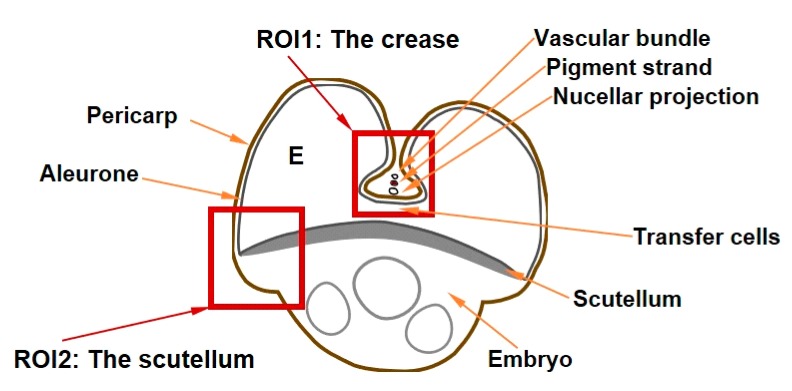
A representative wheat (*Triticum aestivum* L.) grain cross section with the two regions of interest (ROI) highlighted with red squares, namely ROI1 (the crease) and ROI2 (the scutellum). E—endosperm.

Iron species and Fe ligands were studied in two different regions of interests (Figure 3) of the frozen-hydrated grain cross-sections of the four wheat cultivars. The first region of interest comprised nucellar projection, modified aleurone, endosperm, transfer cells, and scutellum. The second region of interest comprised aleurone, scutellum, embryo, endosperm, and pericarp.

To identify higher Fe signal pixels—selected for subsequent micro-XANES analysis—the regions of interest were first subjected to a fast (to avoid photoreduction of Fe by the focused X-ray beam) micro-XRF mapping at the ID21 beamline at ESRF to localise Fe, P, and S (the quantitative maps are shown in Figures S4 and S5). By identifying Fe hotspots, the best signal-to-noise ratio in Fe K-edge micro-XANES spectra was ensured. In endosperm, the concentrations of Fe were too small (on average 3.5 mg·kg^−1^ fresh weight in imbibed grains of awned cultivars and 11.4 mg·kg^−1^ fresh weight in imbibed grains of awnletted cultivars) to yield micro-XANES spectra of sufficient quality. Similarly, larger Fe concentrations were found in aleurone and in pericarp of the awnletted cultivars (82.3 and 24.6 mg·kg^−1^ Fe fresh weight, respectively) than in aleurone of the awned cultivars (49.2 and 12.2 mg·kg^−1^ Fe fresh weight, respectively). By contrast, in embryo and nucellar projection the average Fe concentration of awned cultivars (33.8 and 160 mg·kg^−1^ Fe fresh weight, respectively) was larger than in awnletted cultivars (12.5 and 92 mg·kg^−1^ Fe fresh weight, respectively). In scutellum, both cultivars contained similar Fe concentration (60.5 mg·kg^−1^ Fe fresh weight in awned cultivars and 64 mg·kg^−1^ Fe fresh weight in awnletted cultivars).

**Figure 4:**
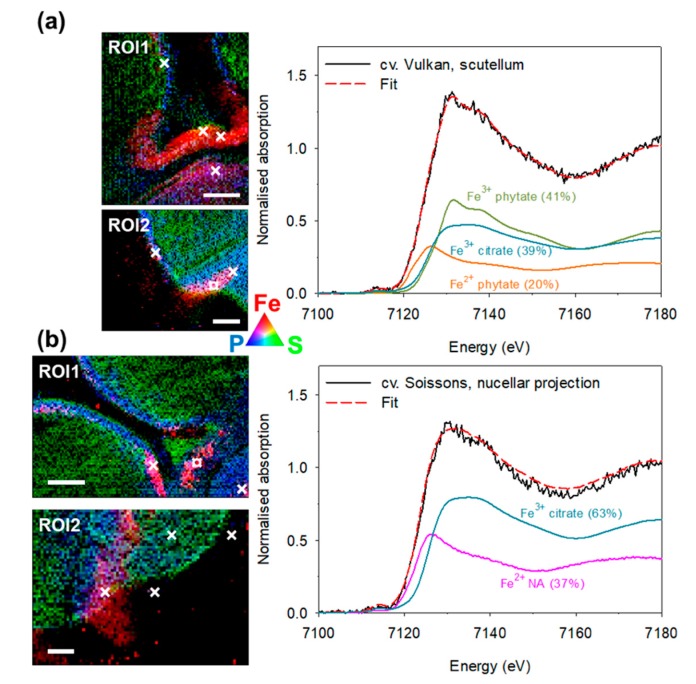
Co-localisation images of iron (Fe) in red, sulphur (S) in green and phosphorus (P) in blue in the two regions of interest (ROI): the crease (ROI1) and the scutellum (ROI2) in the frozen-hydrated grain of wheat (*Triticum aestivum* L.) cultivar Vulkan (**a**) and Soissons (**b**), the awned wheat cultivars. × indicates pixels where Fe K-edge micro-XANES spectra were recorded and ¤ indicates where the selected Fe K-edge micro-XANES spectra (solid line) was recorded and is displayed on the right-hand side. The best linear combination fit (red dashed line) was obtained by the spectra of the reference Fe compounds. The relative amount of each component is given in parentheses. eV—electron volts; NA—nicotianamine. Scale bars = 200 µm. Quantitative distribution maps of Fe, P and S can be found in Figure S4.

**Figure 5:**
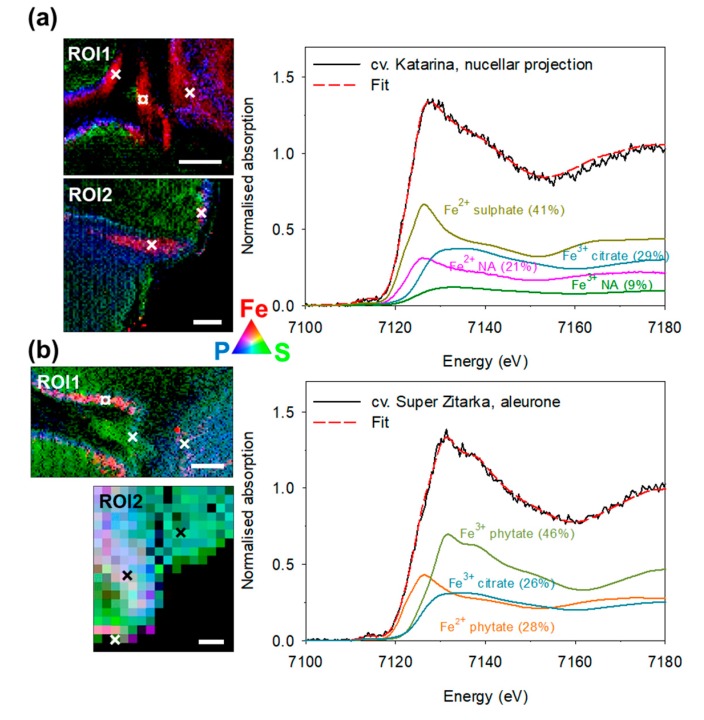
Co-localisation images of iron (Fe) in red, sulphur (S) in green and phosphorus (P) in blue in the two regions of interest (ROI): the crease (ROI1) and the scutellum (ROI2) in the frozen-hydrated grain of wheat (*Triticum aestivum* L.) cultivar Katarina (**a**) and Super Zitarka (**b**), the awnletted wheat cultivars. × indicates pixels where Fe K-edge micro-XANES spectra were recorded and ¤ indicates where the selected Fe K-edge micro-XANES spectra (solid line) was recorded and is displayed on the right-hand side. The best linear combination fit (red dashed line) was obtained by the spectra of the reference Fe compounds. The relative amount of each component is given in parentheses. eV—electron volts; NA—nicotianamine. Scale bars = 200 µm. Quantitative distribution maps of Fe, P and S can be found in Figure S5.

The micro-XANES spectra from selected Fe hotspots (2 to 4 per section as indicated on the Fe, P and S co-localisation maps in Figure 4 and Figure 5) were compared to the spectra of the Fe reference compounds and complexes (Figure S6; reported previously [25,33]).

The Fe K-edge micro-XANES spectra could be described as linear combinations of the Fe K-edge XANES spectra of the following Fe complexes: Fe^2+^ phytate, Fe^2+^ sulphate, Fe^2+^ nicotianamine, Fe^3+^ phytate, Fe^3+^ nicotianamine, Fe^3+^ citrate, and α-Fe^3+^OOH (Fe oxide-hydroxide; goethite). Relative amount of each Fe complex in the combination (Table S2) was obtained from the best fit with a ± 1% error for the Fe^2+^/Fe^3+^ complex ratio and a ± 5% error for the Fe^3+^ phytate/Fe^3+^ non-phytate ratio.

**Figure 6:**
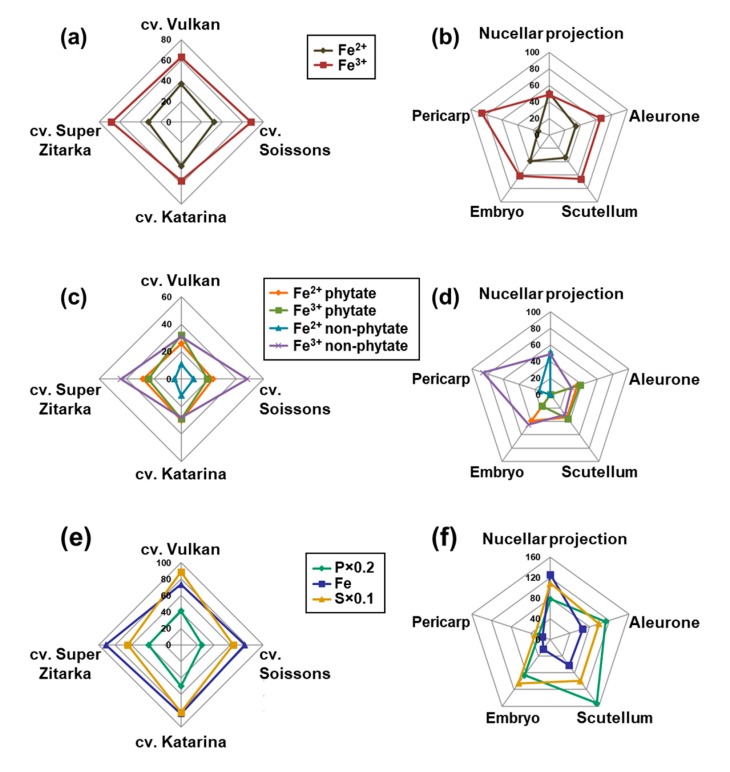
Average relative amounts (%) of iron (Fe) species, Fe ligands and Fe, phosphorus (P) and sulphur (S) concentration in the grain of wheat (*Triticum aestivum* L.) cultivars Vulkan and Soissons (awned wheat cultivars) and Katarina and Super Zitarka (awnletted wheat cultivars) (**a**,**c**,**e**) and in grain tissues (**b**,**d**,**f**). Phosphorus, Fe and S concentrations are in mg·kg^−1^ dry weight (**e**) or fresh weight (**f**). In (**b**,**d**,**f**) results indicate average across all four cultivars.

On average, the four cultivars did not differ in the Fe species and Fe ligand composition (Figure 6a,c). Ferric species was predominant in all four cultivars, with 64% of the total Fe found in this form (Figure 6a), which was a cumulation of 26% being phytate ligand and 38% non-phytate ligands. The remaining Fe was present as ferrous species (36%) in all four cultivars, which was a cumulation of 27% bound to phytate and 9% to non-phytate ligands. In total, 53% of Fe was found bound to phytate and the remaining 47% to non-phytate ligands (Figure 6b and Table S2).

A significant tissue-specificity for Fe speciation and Fe ligand composition was observed (Figure 6b,d). In all tissues studied, the majority of Fe species were ferric—except in nucellar projection, with equal contribution of ferric and ferrous species (Figure 6b). In the order of decreasing content of ferric species, the list of tissues is: pericarp < aleurone = scutellum < embryo < nucellar projection (Figure 6b). By the proportion of phytate ligands, the tissues were ordered as: aleurone < scutellum << embryo << nucellar projection = pericarp, with the latter two tissues having no phytate ligands, but only non-phytate ligands (Figure 6d and Table S2). Of non-phytate ligands in nucellar projection Fe^3+^ citrate was most prominent (34%), followed by Fe^2+^ nicotianamine (29%), Fe^2+^ sulphate (22%) and Fe^3+^ nicotianamine. By contrast, the pericarp contained mostly Fe^3+^ oxide-hydroxide (52%), followed by Fe^3+^ nicotianamine (23%), Fe^2+^ sulphate (14%) and Fe^3+^ citrate (12%). Of non-phytate ligands in other tissues, only Fe^3+^ citrate was found, with the largest proportions in embryo (45%), followed by scutellum (30%) and aleurone (27%). No clear correlation between the total and local Fe, P and S concentration and the Fe ligand profile in tissues could be discerned (Figure 6e,f). The nucellar projection contained the largest concentration of Fe, while pericarp contained the smallest concentration of Fe, P and S (Figure 6f).

## 3. Discussion

The frequent lack in intake of essential mineral elements in human diets can be significantly improved by shaping agronomic practice and/or designing staple crops to generate mineral-dense produce [7]. Agronomic approaches have been efficient when (i) the soil contains insufficient amounts of certain element(s), which can be added to the agricultural system as fertilizers or (ii) when changes to phytoavailability of elements in the rhizosphere are required and pH-related intervention can offer solutions. On the other hand, variability in the elemental composition of the edible produce can be exploited to (i) introduce cultivars with superior mineral-use efficiency, provided there is no penalty to agronomically important traits or (ii) to identify candidate genes for future genetic optimisation [9]. After these interventions are implemented and a produce with the largest possible inherent concentration is available, the Fe status of the individual and other food components (e.g., dietary fibre, organic acids) will still play crucial roles in the availability of a certain element, with Fe being particularly problematic [34], further complicating the efforts to ensure optimal nutrition in humans.

### 3.1. Awned Cultivars Accumulate More Ca and Mn But Less Fe in Grain than Awnletted Wheat Cultivars

We investigated the diversity in grain mineral element accumulation in 20 wheat cultivars and found that there is a link between the awn length and the Ca, Mn and Fe concentrations (Figure 1). In wheat, awns have been shown to have transpiration and photosynthetic activity [20], thus their presence could contribute to the translocation of elements taken up by roots on the one side and/or to the phloem-driven (re)allocation of assimilates on the other side, thereby affecting mineral element density in the grain. This connection has, however, not been investigated so far. Awnletted wheats have been shown to produce significantly more grains per unit area and per spike, resulting in a significant reductions in grain size and an increased frequency of small, shrivelled grains [35]. Our observations did not fully support this report, since there was no significant difference in 1000-grain weight between the awned and awnletted cultivars, both for the established agronomic values and for those from our experiment (Table S1). The observed differences in elemental concentrations are conceivably not a consequence of dilution by grain weight, but rather arise from genetic differences in uptake, allocation and/or mobilisation or Ca, Mn and Fe in these cultivars. It may however be, that by accumulating larger Fe concentration in the embryo and particularly in the nucellar projection in awned cultivars compared to awnletted cultivars as observed in our study, less Fe is being translocated to other grain tissues, resulting in larger total Fe concentration in the whole grain.

On average, total Fe concentrations in grain from our experiment showed 2.03-fold variability (Figure 2), which is in a similar range as the value 1.76 reported for 150 bread wheat cultivars [14]. The whole grain Fe concentrations in all studied cultivars exceed the reported maximum value (50.8 mg Fe·kg^−1^) for the bread wheat cultivars [14], but were within the range of observations in spelt (*Triticum spelta* L.) grain, for which up to 99 mg Fe·kg^−1^ was found, but with significant variation due to the year and the location [36]. Iron concentration in barley grain exceeding 100 mg·kg^−1^ has also been reported [37].

The positive correlation between P and Fe concentrations was observed (Figures S2 and S3) in agreement with findings in bread wheat [14] and in spelt [36], suggesting that the increased Fe concentration may be accompanied by a decreased bioavailability (i.e., due to phytate), presenting a further challenge for biofortification. However, the positive relationship has not been observed in all instances [38,39] and a strong genotype, environment and/or genotype × environment interaction has been shown to affect the relationship [40,41]. The positive correlation between grain Fe and Zn observed also in our experiment (Figures S2 and S3), seems to be quite stable as it has been consistently reported, over different seasons and locations, for example in wheat [14,38], durum wheat [39], spelt [36], and barley [15,37]. It could be attributed to the limited specificity of transporters and metal ligands for either Zn or Fe [42], suggesting that the increased density could be achieved simultaneously for a larger number of trace elements. Still, the issues with the bioavailability of these trace elements will have to be addressed before any such observations are implemented into breeding strategies.

### 3.2. Iron Speciation and Iron Ligands in Wheat Grain Are Stable across Cultivars Differing in Total Iron Concentration

To complement the current knowledge on Fe speciation and Fe ligands in whole wheat grain [25] and some of its tissues [28] we studied these traits in five grain tissues of four wheat cultivars. Initial X-ray fluorescence mapping in frozen hydrated cross-sections of the grain revealed Fe hotspots and provided information on P and S distribution as well. Based on the co-localisation of Fe, P, and S (Figure 4 and Figure 5), selected regions of interest (Figure 3) were easily distinguished and high-Fe pixels were investigated by micro-XANES. There was no apparent difference in the Fe speciation or Fe ligands in the grain of awned and awnletted cultivars, regardless of the differences in the Fe concentrations (Figure 6a,c) indicating that the total Fe concentration in the grain does not influence Fe species or Fe ligands. Similar results were found by Singh et al. [25], who included in the analysis a wild relative of common wheat, *Aegilops kotschyi* Boiss., which contains up to three times larger Fe concentrations than grain of wheat landraces. The relative amounts of ferrous (36%) species in wheat grain—up to one third of total Fe as assessed by micro-XANES in our experiment (Figure 4, Figure 5 and Figure 6)—was somewhat larger than in previous findings in whole wheat with values between 14% and 24% [25]. Furthermore, on average 53% of Fe was bound to phytate. The proportion of non-phytate ligands is in agreement with another study [28] on wheat and Tartary buckwheat grain, where 22% of total Fe was bound to non-phytate ligands [33]. Perplexingly, no direct association of the Fe ligand profile could be found with Fe bioavailability (assessed using Caco-2 cell system) in Tartary buckwheat sprouts containing a much larger proportion (55%) of Fe^3+^ citrate [33]. In legume seeds, which store large amounts of Fe in ferritin, progressive accumulation of phytate with seed maturity limits Fe bioavailability, as demonstrated by comparing immature and mature pea (*Pisum sativum* L.) seeds [43]. Apparently, more studies are required to reach consensus on the connection between Fe ligands and Fe bioavailability from different plant-based sources.

### 3.3. Distinct Tissue Specificity in Iron Speciation, Iron Ligands and Iron Concentration in Wheat Grain

At the tissue level a large variability in the Fe speciation and Fe ligands was found (Figure 6) in line with a previous report [28], suggesting differences in bioavailability of Fe from different grain tissues. All tissues contained >60% of ferric species in line with analyses in whole grain [25]. The only exception was nucellar projection, where equal amounts of Fe^3+^ and Fe^2+^ were found (Figure 6b). Regarding the Fe ligands, the presence of non-phytate ligands in the pericarp and nucellar projection was particularly striking (Figure 6d). The pericarp is a maternal tissue and is typically accessed by xylem, which may be a reason for the Fe-citrate pool. There is some evidence that Fe from the Tartary buckwheat pericarp [33] and from the wheat bran [44] is relatively bioavailable. While Tartary buckwheat pericarp is not edible, the inclusion of wheat bran in the meal would, despite markedly increased phytate concentration, outweigh the negative effect of phytate-induced losses in bioavailability.

The major part of cereal grain loading with micro and macronutrients is presumed to take place through vasculature, which in mature grain is shrunk and borders the pigment strand, which in turn borders the nucellar projection (Figure 3). During grain maturity the tissues within the crease undergo a series of transformations, and nutrients passing into the endosperm must cross the pigment strand, the nucellar projection, and the endosperm transfer cells [45]. The nucellar projection is a part of the nucellar tissue that faces the vascular tissue, has a morphology characteristic of transfer cells [46] and contains large concentration of Fe, while the pigment strand was rich in Mn, making these two tissues clearly distinguishable [12,25,32]. As already follows from the apparent lack of co-localisation of Fe and P in the nucellar projection (Figure 4 and Figure 5; [12,25]) only non-phytate ligands were found there. Our analysis also confirms the presence of nicotianamine in the nucellar projection reported previously in [28]. For the first time, ferric and ferrous nicotianamine compounds are demonstrated to exist in a grain tissue (together 45% of Fe ligands). In addition, some Fe was bound to sulphate (22%), in agreement with previous reports [28] and predicted from S localisation in this tissue (Figure 4, Figure 5 and Figure 6, Figures S4 and S5) and exclusion of P [25].

In embryo, scutellum and aleurone, the only non-phytate ligand found was citrate, which is in agreement with results for cotyledons in Tartary buckwheat grain [33]. Most of the Fe present in the liquid endosperm in pea was found as a mixture of Fe^3+^ citrate and malate [47]. The mixture has been shown to undergo an ascorbate-driven reduction [47] which makes Fe more mobile within the developing seedling. Ferric citrate-malate complexes have been demonstrated as the main form of Fe circulating in pea (*Pisum sativum* L.) plants [19]. In pea, the mother plant transports Fe^3+^ malate/citrate complexes via the seed coat to the embryo, which in turn secretes ascorbate to reduce Fe^3+^ to Fe^2+^ for uptake during germination [47]. These observations suggest active participation of all grain tissues and not only crease tissues in Fe loading of the grain and seed.

## 4. Conclusions

Because awns presumably have photosynthetic activity, awned and awnletted wheat cultivars were compared for their 1000-grain weight, mineral composition and Fe speciation and Fe local chemical environment. While there was no difference in 1000-grain weight, a larger Ca and Mn, but smaller Fe concentrations, were found in whole grain of awned than in awnletted cultivars. Genetic and/ or metabolic reasons behind the observed differences in mineral composition will need to be studied in future experiments. The evaluation of Fe speciation and Fe ligands revealed that differences in total Fe concentration were not accompanied by differences in Fe speciation (on average 64% of Fe existed as ferric and 36% as ferrous species) or Fe ligands (on average 53% were phytate and 47% were non-phytate ligands) in the two awned and two awnletted cultivars studied using micro-XANES. Contrastingly, there was a distinct tissue-specificity with pericarp containing the largest proportion (86%) of ferric species and nucellar projection (49%) the smallest. Iron was predominantly bound to phytate in aleurone, scutellum and embryo (72%, 70%, and 56%, respectively), while in nucellar projection and pericarp Fe was bound only to non-phytate ligands. Assuming Fe bioavailability depends on Fe ligands, we conclude that Fe bioavailability from wheat grain is tissue specific.

## 5. Materials and Methods

### 5.1. Plant Material and Total Element Concentration in Grain

The grain of 20 different wheat (*Triticum aestivum* L.) cultivars were obtained from the Agricultural Institute of Slovenia, with their agronomic characteristics reported in Table S1. Among these wheat cultivars, nine were awned (cv. Euclide, cv. Lukullus, cv. Vulkan, cv. Isengrain, cv. Renan, cv. Soissons, cv. Ingenio, cv. Bologna, and cv. Element) and 11 were awnletted (cv. BC Nina, cv. BC Renata, cv. Rosario, cv. Felix, cv. Renata, cv. Katarina, cv. BC Zdenka, cv. Anđelka, cv. Gracia, cv. Bastide, and cv. Super Zitarka). The plants were grown in the field of the Infrastructure centre Jablje (central Slovenia: 46°8′59″ N, 14°33′31″ E, 307 m above sea level) in 2014/2015 on pseudogley-gley soil type, which has a silty clay texture. The previous crop on the field site was grain maize (*Zea mays* L.). The field was fertilised with 205 kg·ha^−1^ N (in 5 rations), 90 kg·ha^−1^ P_2_O_5_ and 120 kg·ha^−1^ K_2_O; 400–750 germinative seeds m^−2^ were sowed respecting interrow spacing of 12.5 cm. Trial layout was a randomized block design with four repetitions, with each plot having 7.5 m^2^. At maturity, the plants were harvested, the 1000-grain weight was determined (g), the grain was homogenised and milled or stored for the localisation analyses. The ground material was pressed into pellets (6–12 for each cultivar) using a pellet die and hydraulic press. Total concentrations of P, S, K, Ca, Mn, Fe, and Zn were measured in whole grain samples and in standard reference material NIST SRM 1573a (tomato leaves) for quality assurance using X-ray fluorescence, as described previously [48]. Based on the total Fe concentration in the whole grain of the 20 wheat cultivars, four contrasting cultivars were selected. Cultivars Katarina and Super Zitarka, the two awnletted wheat cultivars, had a larger total Fe concentration than the two awned cultivars Vulkan and Soissons.

### 5.2. Sample Preparation and Micro-XRF Mapping and Micro-XANES Analysis

The grain of the four wheat cultivars was soaked in MiliQ water for 2 h at 4 °C. Whole grain was hand-cut transversely at the embryo location under a stereomicroscope into approximately 200 µm thick sections using new stainless-steel platinum-coated razor blades and frozen immediately in liquid isopentane (Sigma-Aldrich, St. Louis, MO, USA) cooled by liquid nitrogen [49]. Frozen-hydrated sections were sandwiched between two Ultralene^®^ foils (each 4 µm thick), deposited on custom made Cu holders and analysed at the ID21 beamline (ESRF, Grenoble, France) as described previously [33]. In short, measurements of Fe K-edge micro-XANES (recorded in the energy region from 7040 to 7250 eV) were performed on high Fe pixels (*n* = 2–9) identified by fast mapping of grain sections using synchrotron radiation micro-X-ray fluorescence (micro-XRF) at the same beamline. This approach enabled the best quality micro-XANES spectra of the two regions of interest in the wheat grain: the crease and the scutellum. Specific attention was paid to avoid radiation damage and photoreduction of Fe^3+^ [50], as described in detail in [25,33].

The qualitative distribution maps of Fe, P, and S were quantitatively analysed, as described previously [51,52] and the quantitative distribution maps and co-localisation maps were generated with PyMCA software [53]. The Fe K-edge micro-XANES spectra were analysed with the IFEFFIT program package Athena [54], where linear combination fitting was performed using the following reference Fe^2+^ and Fe^3+^ complexes: Fe^2+^ phytate, Fe^2+^ nicotianamine, Fe^2+^ sulphate, Fe^3+^ phytate, Fe^3+^ nicotianamine, Fe^3+^ citrate and α-Fe^3+^OOH - oxide/hydroxide (goethite). The preparation and analysis of these reference Fe complexes has been described elsewhere [25,33], except for Fe nicotianamine standards, which were synthesized by mixing water solution of nicotianamine (CAS 34441-14-0, Santa Cruz Biotechnology, Inc., USA) with FeCl_2_ × 4H_2_O (Sigma-Aldrich) or FeCl_3_ × 6H_2_O (Sigma-Aldrich) water solution at the molar ratio of 10:1 in water. Final pH was adjusted to 6.5–7.0. XANES spectra of the reference compounds Fe^2+^ nicotianamine and Fe^3+^ nicotianamine were measured at XAFS beamline of synchrotron Elettra (Trieste, Italy) in transmission detection mode, on homogeneous pellets with optical thickness of about 2 above Fe absorption K-edge. Iron species and Fe ligands for each wheat cultivar were obtained by averaging across all tissue-specific XANES results in each cultivar.

### 5.3. Statistical Analysis

Pairwise comparisons were tested by Student *t*-test at *p* < 0.05 and linear regression analysis was performed in SigmaPlot version 13.0 (Systat Software, San Jose, CA). Clustering and heatmap was created using z-transformed average total concentrations in whole grain using function heatmap.2 in gplots package within R i386 3.0.2 software.

## Supplementary Materials

The following are available online at https://www.mdpi.com/2223-7747/9/1/79/s1. Figure S1: Hierarchical clustering and heatmap of grain elemental composition of all wheat cultivars studied. Figure S2: Correlations between element concentrations in whole grain for all wheat cultivars studied. Figure S3: Correlations between element concentrations in whole grain for the selected four wheat cultivars. Figure S4: Quantitative distribution maps in the grain of awned wheat cultivars. Figure S5: Quantitative distribution maps in the grain of awnletted wheat cultivars. Figure S6: Iron K-edge XANES of reference compounds used in the linear combination fitting. Table S1: Agronomic characteristics of the wheat cultivars studied. Table S2: Relative amounts of iron ligands in the grain wheat cultivars and in different grain tissues.
