# Supplementary material for: Mineral Element Composition in Grain of Awned and Awnletted Wheat (Triticum aestivum L.) Cultivars: Tissue-Specific Iron Speciation and Phytate and Non-Phytate Ligand Ratio
Source: Plants (Basel). 2020 Jan 8;9(1):79. doi: 10.3390/plants9010079 (PMC7020463; doi:10.3390/plants9010079)
Supplement: Supplementary file 1 [file plants-09-00079-s001.pdf]

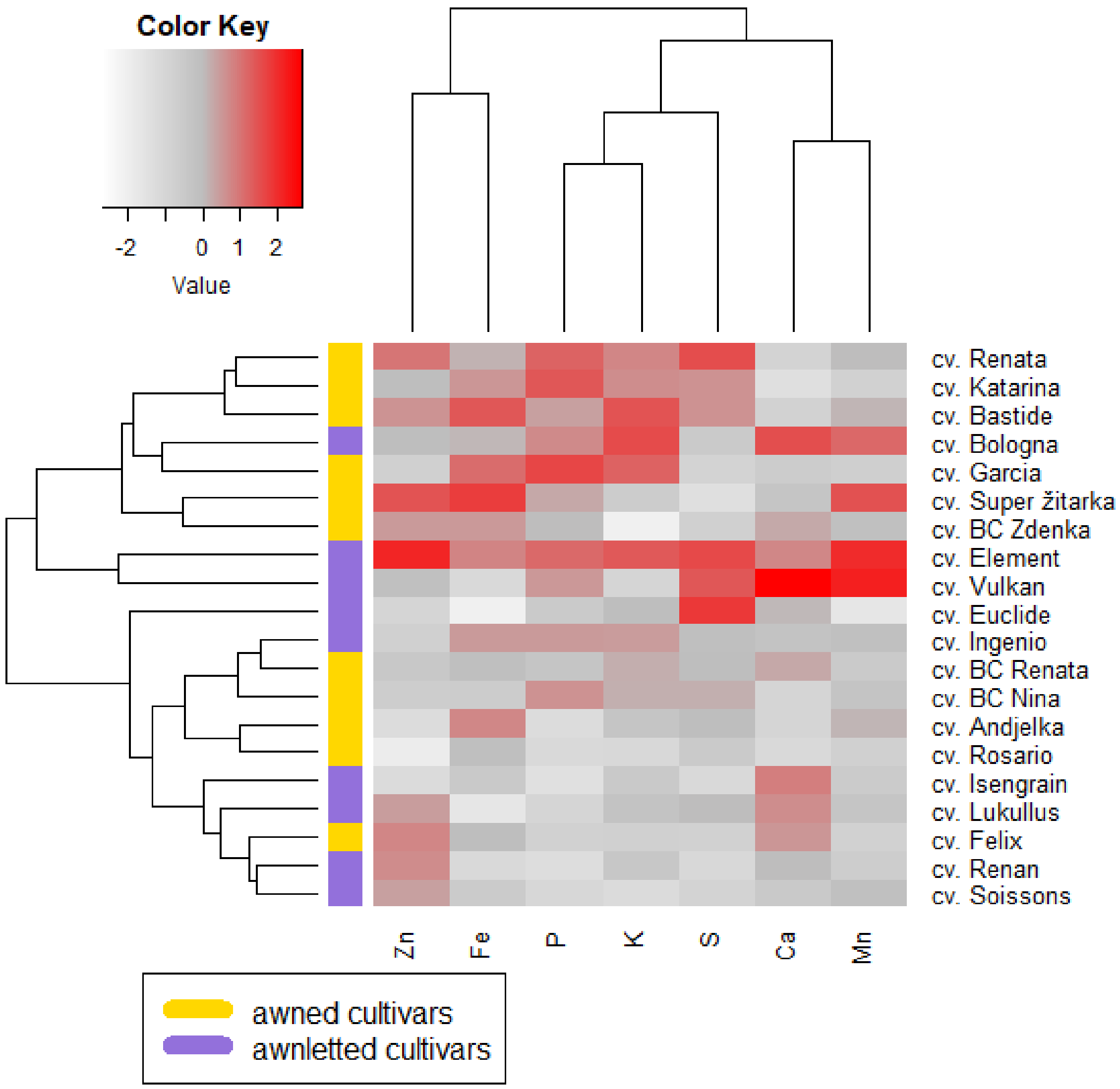

Figure S1. Hierarchical clustering and heatmap constructed by two-way clustering of z-transformed total zinc (Zn), iron (Fe), phosphorus (P), potassium (K), sulphur (S), calcium (Ca) and manganese (Mn) concentrations in whole grain of wheat (*Triticum aestivum* L.) cultivars differing in the awn type (awned cultivars have long awns and awnletted cultivars have short or no awns) grown in the same field.

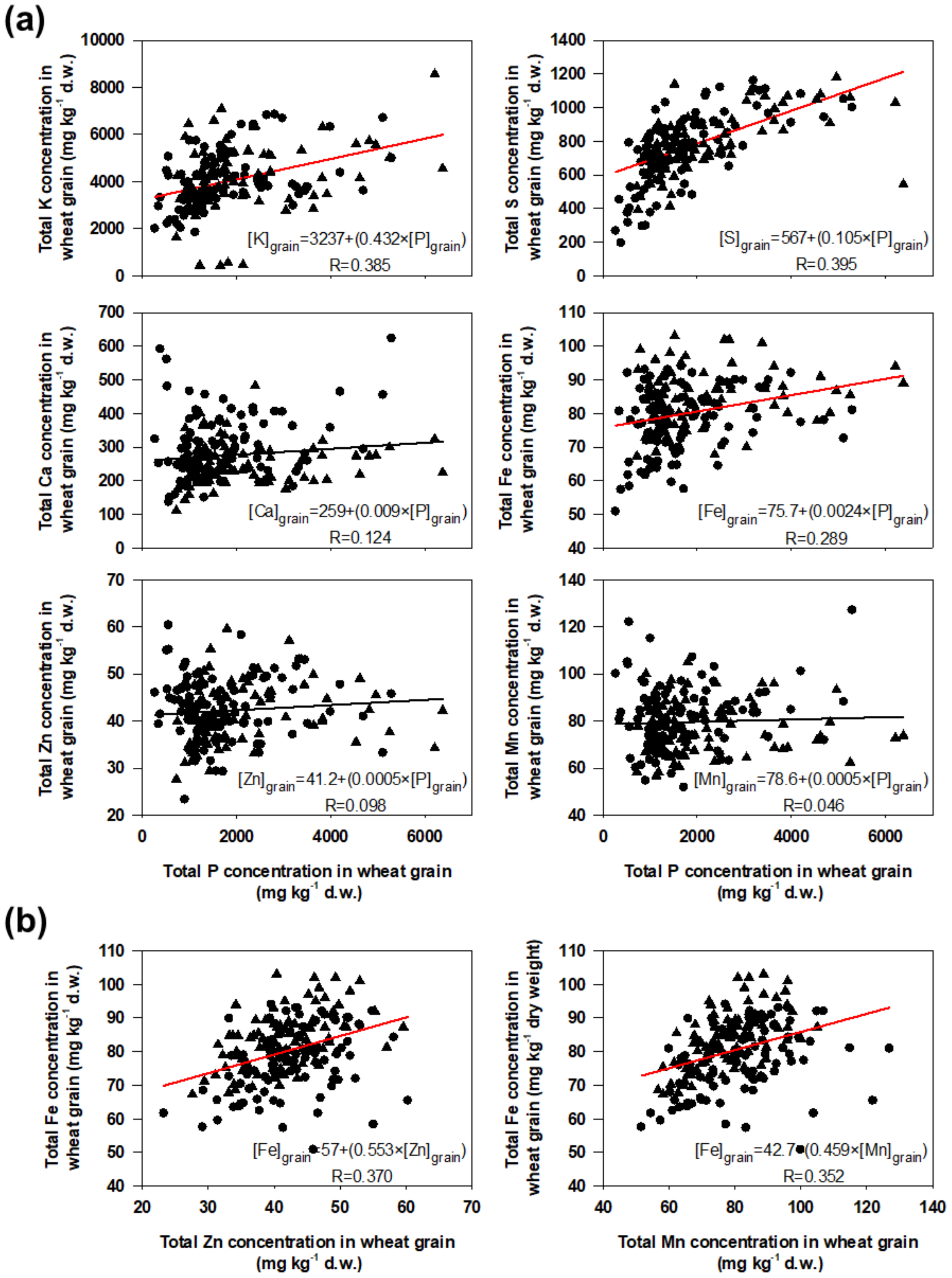

Figure S2. Linear regression relationships between total phosphorus (P) concentration and potassium (K), sulphur (S), calcium (Ca), iron (Fe), zinc (Zn) or manganese (Mn) concentration (**a**) and between Zn and Fe, and Mn and Fe (**b**) in the whole grain of twenty wheat (*Triticum aestivum* L.) cultivars differing in the awn type (awned cultivars have long awns and awnletted cultivars have short or no awns) grown in the same field. Circles represent awned cultivars and triangles represent awnletted cultivars. Significant relationships ($P < 0.001$) are highlighted in red. Note that graphs show data range and for clarity purposes do not begin with zero in all cases; d.w., dry weight.

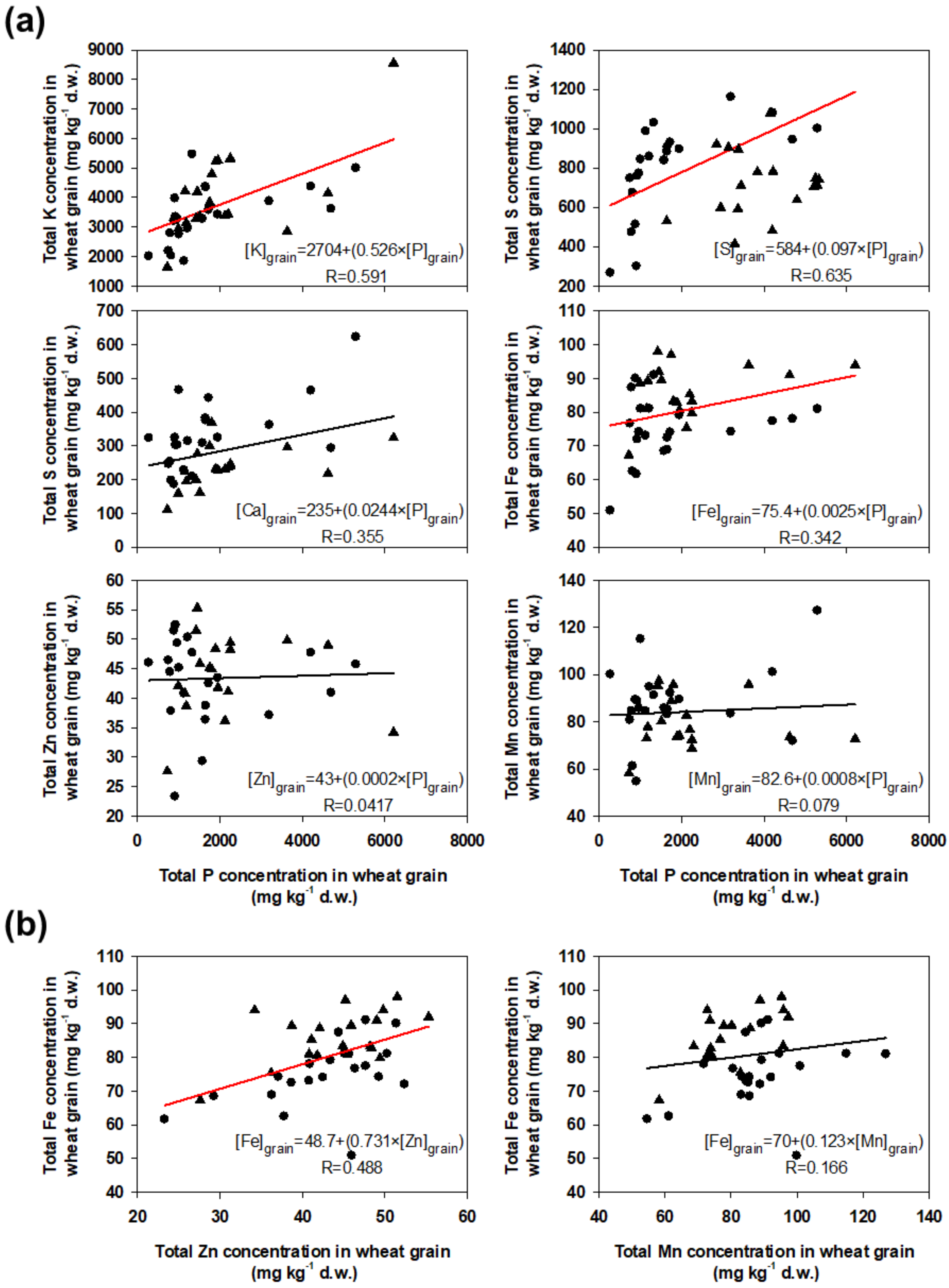

Figure S3. Linear regression relationships between total phosphorus (P) concentration and potassium (K), sulphur (S), calcium (Ca), iron (Fe), zinc (Zn) or manganese (Mn) concentration (**a**) and between Zn and Fe, and Mn and Fe (**b**) in the whole grain of the selected four wheat (*Triticum aestivum* L.) cultivars differing in the awn type (awned cultivars have long awns and awnletted cultivars have short or no awns) grown in the same field. Cultivars Vulkan and Soissons are awned cultivars (represented by circles) and cultivars Katarina and Super Zitarka are awnletted cultivars (represented by triangles). Significant relationships (P <.0.001) are highlighted in red. Note that graphs show data range and for clarity purposes do not begin with zero in all cases; d.w., dry weight.

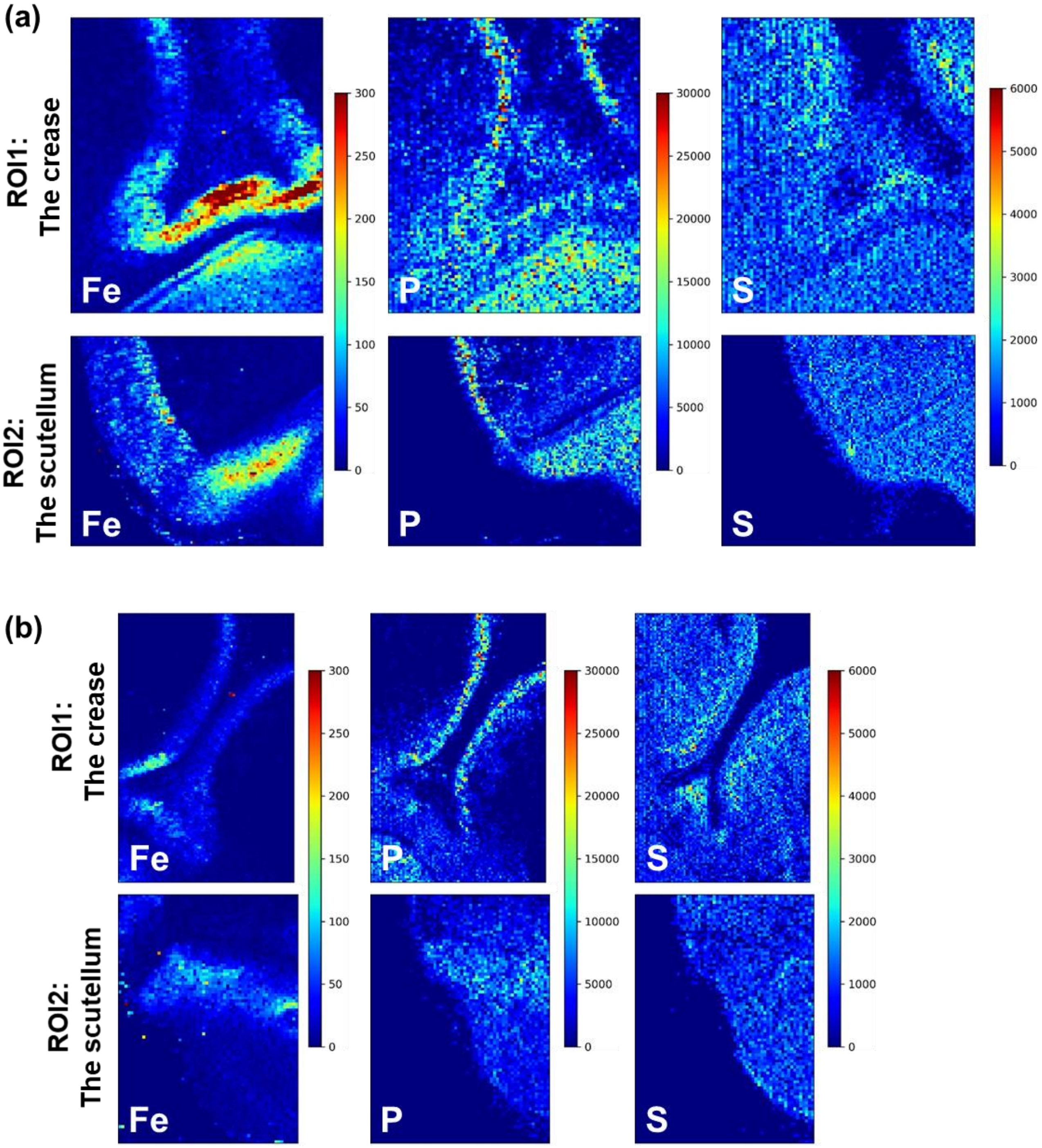

Figure S4. Quantitative distribution maps of iron (Fe), phosphorus (P) and sulphur (S) of the two regions of interest (ROI): the crease and the scutellum in the frozen-hydrated grain of two awned (having long awns) wheat (*Triticum aestivum* L.) cultivars Vulkan (**a**) and Soissons (**b**). Colour legends indicate concentration in mg kg^{-1} fresh weight.

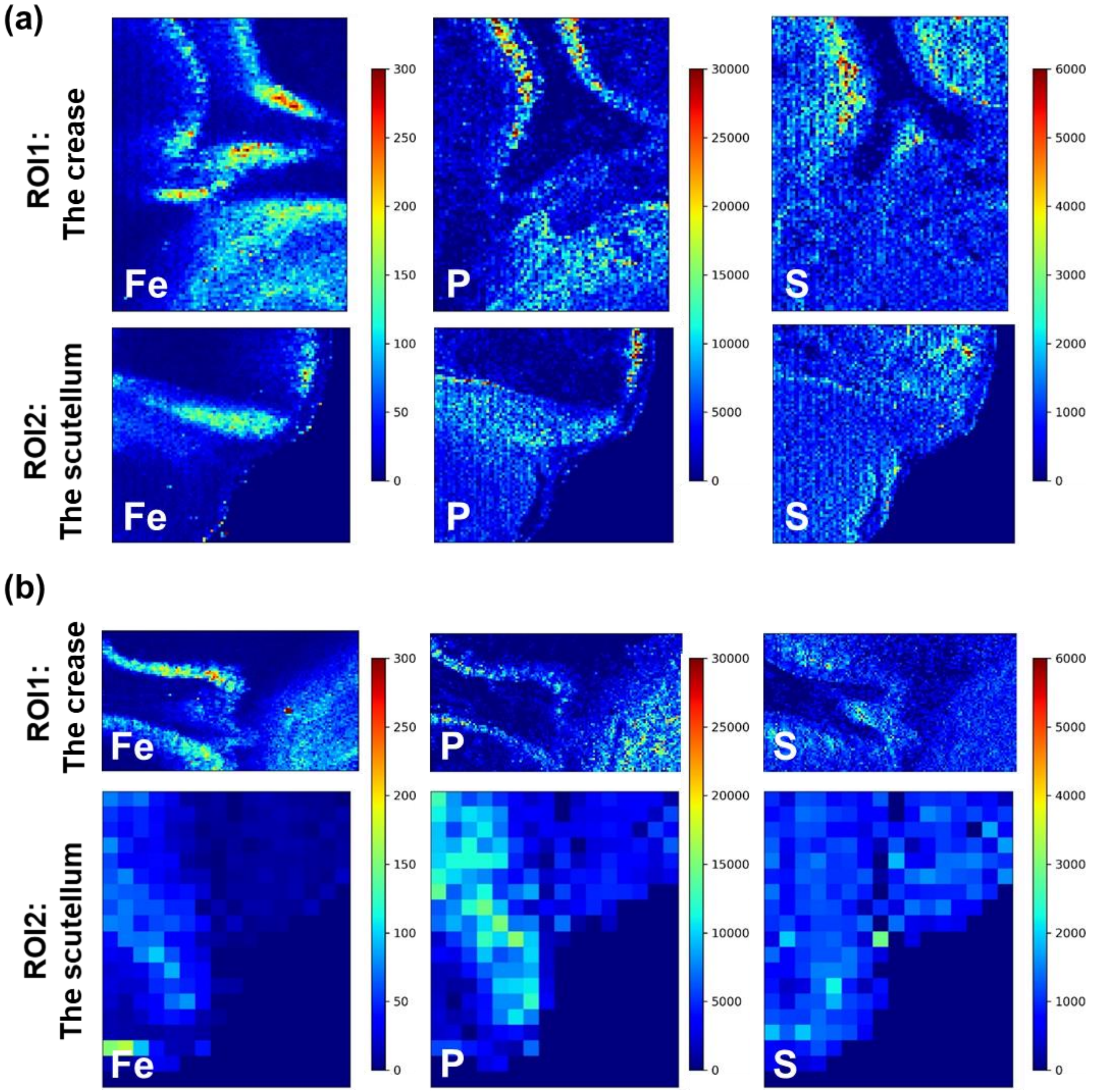

Figure S5. Quantitative distribution maps of iron (Fe), phosphorus (P) and sulphur (S) of the two regions of interest (ROI): the crease and the scutellum in the frozen-hydrated grain of two awnletted (having short or no awns) wheat (*Triticum aestivum* L.) cultivars Katarina (**a**) and Super Zitarka (**b**). Colour legends indicate concentration in mg kg^{-1} fresh weight.

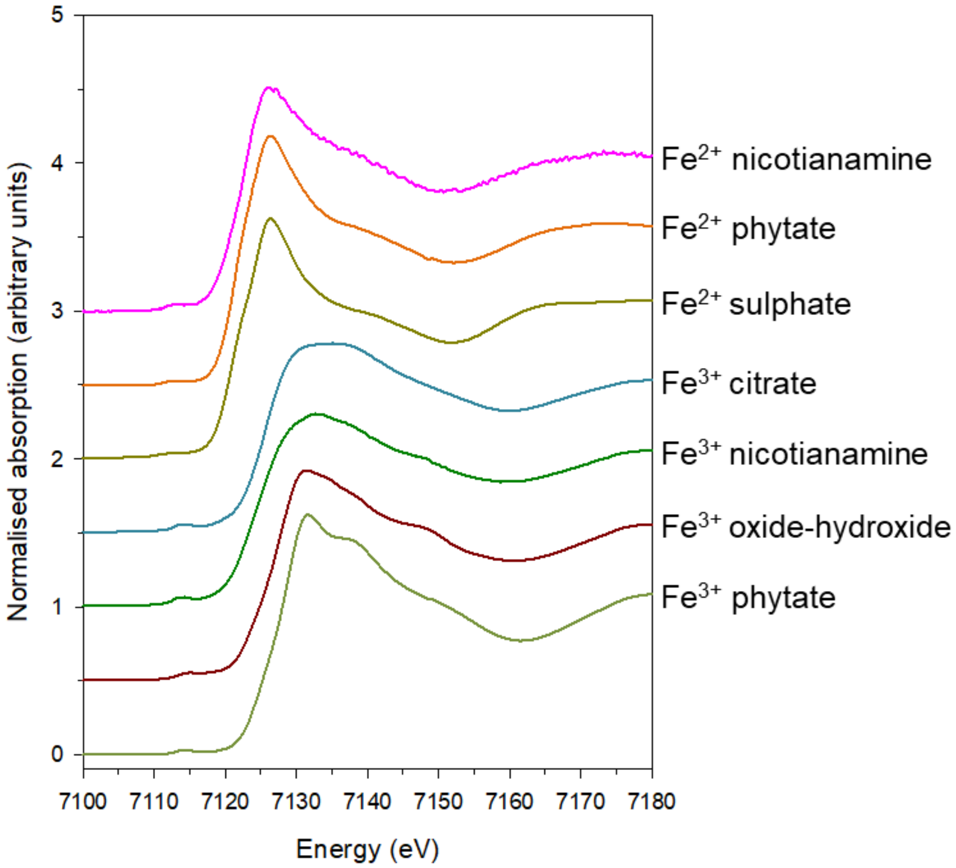

Figure S6. Iron (Fe) K-edge XANES spectra of the reference compounds used in linear combination fitting. Spectra are shifted vertically for clarity. eV, electron volts.

Table S1. Agronomic characteristics of the wheat (*Triticum aestivum* L.) cultivars studied (**a**) and averages for awned and awnletted cultivars (**b**).

(a)

Wheat (*Triticum aestivum* L.) Cultivar	Awn Type	1000-grain Weight (g)	Total Protein Content (%)	Sedimentation Index (mL)	Quality Index (A, B1, B2)	1000-grain Weight (g)**
**Bologna***	Awned	35	14.2	66	A	35.2
**CCB - Ingenio***	Awned	54	13.5	57	B2	54.1
**Element**	Awned	n.d.	n.d.	n.d.	B1/A	44.7
**Euclide***	Awned	46	12.4	43	B2	47.4
**Isengrain***	Awned	41	11.9	39	B2	42.2
**Lukullus§**	Awned	40	13.3	42	B1	48.4
**Renan***	Awned	49	12.9	47	B1	51.5
**Soissons***	Awned	39	12.5	41	B2	41.6
**Vulkan***	Awned	38	14	50	B1	38.5
**Anđelka¥**	Awnletted	39	13	41	n.d.	41.7
**Bastide***	Awnletted	41	12	37	B2	47.9
**BC Nina***	Awnletted	46	11.7	41	B2	46.5
**BC Renata***	Awnletted	38	13.1	49	B1	44

**BC Zdenka***	Awnletted	34	14.4	57	A	37.4
**Felix***	Awnletted	41	14.4	61	A	43.5
**Garcia***	Awnletted	42	11.9	29	*	49.2
**Katarina¥**	Awnletted	37	12.9	45	B2	42.2
**Renata¥**	Awnletted	40	14.2	52	n.d.	46.1
**Rosario***	Awnletted	41	11.5	38	*	48.5
**Super Zitarka***	Awnletted	44	13.6	42	B1	47.6

(b)

	**1000-grain weight (g)**	**SD**	**Total Protein Content (%)**	**SD**	**Sedimentation Index (mL)**	**SD**	**1000-grain Weight (g)****	**SD**
**Awned cultivars**	42.8	6.36	13.1	0.81	48.1	9.26	44.8	6.12
**Awnletted cultivars**	40.3	3.29	13.0	1.09	44.7	9.35	45.0	3.58

n.d., no data.

Sources:
*http://uvhvvr.arhiv-spletisc.gov.si/fileadmin/uvhvvr.gov.si/pageuploads/OBJAVE_ZA_JAVNOST/Objave_SEME/Sortna_lista/OSL_Psenica_2013.pdf
¥https://www.poljinos.hr/proizvodi-usluge/psenica-jecam/psenica/
§https://www.yumpu.com/xx/document/read/24301120/lukullus-a-b-semenarna-ljubljana
**Our experiment

Table S2. Average relative amounts (%) of iron (Fe) ligands in the grain of wheat (*Triticum aestivum* L.) cultivars: Vulkan and Soissons (awned cultivars) and Katarina and Super Zitarka (awnletted cultivars, (**a**)) and in grain tissues (n = 2–9) (**b**).

(a)

**Wheat Cultivar**	**Relative Amount (%)**			
	**Fe^{2+} phytate**	**Fe^{3+} phytate**	**Fe^{2+} non-phytate**	**Fe^{3+} non-phytate**
**cv. Vulkan**	26	32	11	31
**cv. Soissons**	23	19	9	48
**cv. Katarina**	30	29	12	28
**cv. Super Zitarka**	28	24	5	44

(b)

**Grain Tissue**	**Relative Amount (%)**			
	**Fe^{2+} phytate**	**Fe^{3+} phytate**	**Fe^{2+} non-phytate**	**Fe^{3+} non-phytate**
**Nucellar projection**	0	0	51	49
**Aleurone**	34	38	0	27
**Scutellum**	34	36	0	30
**Embryo**	39	17	0	45
**Pericarp**	0	0	14	86
